# NASA LEAN TEST DETECTS POSTURAL ORTHOSTATIC TACHYCARDIA IN CHRONIC POST-COVID

**DOI:** 10.2340/jrm.v58.45865

**Published:** 2026-07-02

**Authors:** Mario SIEBLER, Andrea MAIER, Stephan THEISS, Sarah BERGÉ, Anna BITTNER, Grit SCHIEFELBEIN, Jürgen WAGNER, Dominik RAAB

**Affiliations:** 1 Institute of Rehabilitation Research, MediClin, Offenburg; 2 Department of Neurology, University Hospital RWTH Aachen, Aachen; 3 Institute of Clinical Neuroscience and Medical Psychology, Medical Faculty and University Hospital Düsseldorf, Heinrich Heine University Düsseldorf, Düsseldorf; 4 Department of Neurology, Medical Faculty and University Hospital Düsseldorf, Heinrich Heine University Düsseldorf, Düsseldorf; 5 Chair for Mechanics and Robotics, University of Duisburg-Essen, Duisburg, Germany

Diagnosis and treatment of patients with post-/long-COVID syndrome (PCS) remain challenging due to frequent, long-lasting, nonspecific, and multifocal symptoms such as fatigue, post-exertional malaise, cognitive impairment, sleep disturbances, and depression leading to long-lasting work inability (1). In patients with PCS, orthostatic intolerance (OI) has been reported in up to 40% of cases (2, 3). One possible manifestation of OI following infectious disease is postural orthostatic tachycardia syndrome (POTS) (4). OI may influence both rehabilitation strategies and clinical outcomes as suggested in previous work on autonomic disorders (5, 6). However, data on the prevalence, characteristics, and clinical relevance of OI in rehabilitation patients with chronic PCS remained limited. To address this knowledge gap, the present study investigated the haemodynamic response to standing up from a supine position by means of the NASA Lean Test (NLT), and examined its relationship to self-reported symptoms in patients with confirmed chronic PCS undergoing inpatient rehabilitation.

## SUBJECTS AND METHODS

**Fig. 1:**
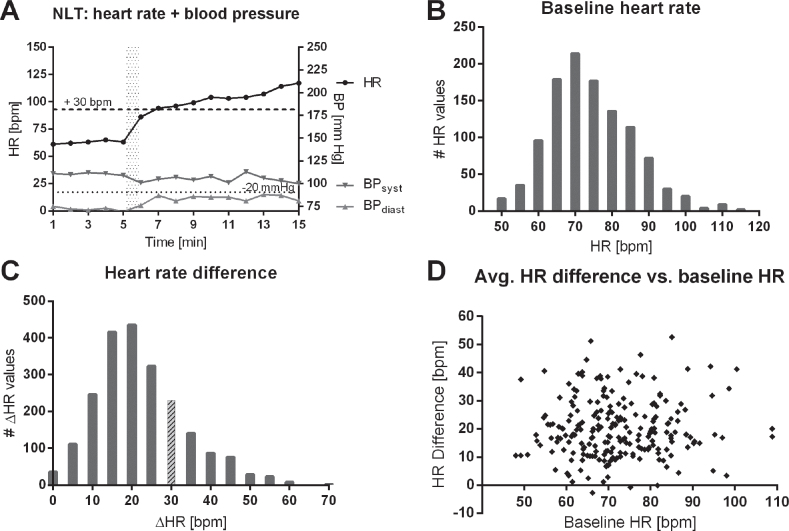
NASA Lean Test: distributions of heart rate and difference. NASA Lean Test (NLT): (A) heart rate (HR, black, left axis), systolic and diastolic blood pressure (BP, grey, right axis) during resting and after standing up (indicated by dotted vertical bar). The dashed line indicates the threshold of 30 beats/min above average resting heart rate. The dotted line indicates systolic blood pressure threshold of 20 mmHg below average resting phase value. In this example, the POTS test is positive with 9 out of 10 HR values above threshold. (B) Resting (baseline) heart rate of 1,105 single measurements in 221 PCS patients. (C) Heart rate difference (2,164 single measurements) to baseline after standing up revealed a wide distribution. The threshold of 30 beats/min is indicated by the hatched bar. (D) Heart rate differences were not correlated with baseline heart rates.

Patients admitted to inpatient rehabilitation for post-/long-COVID syndrome (aged 18–69 years), who had a disease duration of more than 12 months (mean 25.9, SD 15.8) were included in the study. PCS was confirmed according to international classification criteria (1). Patients were screened using a standardized supine-to-standing test (NASA Lean Test, NLT [7]). Heart rate (HR) and blood pressure (BP) as well as symptoms were recorded at 1-min intervals during a 5-min resting supine phase followed by 10 min of standing. The NLT results were evaluated according to established haemodynamic diagnostic criteria by 2 independent experts (5) and by a published algorithm based on the following criteria: at least 2 consecutive ≥ 30 beats, without a decrease in systolic blood pressure greater than 20 mmHg within the first 3 min of standing (7) (Fig. 1A).

The burden of PCS symptoms was assessed using a structured questionnaire. Symptom frequency (never, rarely, often) and intensity (scale 1–10) were asked for fatigue, autonomic symptoms, post-exertional intolerance, dyspnoea/cough, cognitive impairment, pain, acoustic and visual overstimulation, sleep disturbance, and depressive symptoms.

The diagnosis of PCS was confirmed according to international definitions (1, 8) by an independent interdisciplinary expert board consisting of at least 3 physicians. All patients underwent a comprehensive clinical examination by both a neurologist and an internist. Laboratory testing excluded anaemia and thyroid dysfunction. A standard 12-lead electrocardiogram (ECG) was performed to rule out atrial fibrillation or other clinically relevant cardiac arrhythmias.

All participants provided written informed consent. The study was approved by the local ethics committee (Ärztekammer Nordrhein 2025228) and registered in the WHO International Clinical Trials Registry Platform (https://trialsearch.who.int).

## RESULTS

A total of 221 patients with PCS (mean age 47 /SD 12) years; 68.8% female) were included in the study. The mean resting supine HR was 72 (SD 11) bpm (Fig. 1B). After standing, HR increased on average by 20 (SD 10) bpm (Fig. 1C). No correlation was observed between resting HR and the magnitude of HR increase during standing (Fig. 1D).

Overall, 45/221 (20%) of patients fulfilled the haemo-dynamic algorithm criteria for a positive NLT (at least 2 consecutive HR values exceeding the diagnostic threshold of an increase ≥ 30 bpm, no systolic blood pressure decrease over 20 mmHg within the first 3 min of standing) and reported orthostatic symptoms during the test. Most patients had more than 2 values above the diagnostic threshold. The distribution of values ≥ 30 bpm during the 10-min standing period was (noted as values/number of subjects): 0/161; 1/15; 2/4; 3/6; 4/4; 5/8; 6/3; 7/4; 8/6; 9/7; 10/3). Notably, however, 83% of NLT-negative PCS patients reported orthostatic symptoms during the test despite the absence of any BP decrease. The 2 experts separately evaluated the NLT results and revealed an initial inter-rater agreement of 91%. After case-by-case discussion, unanimous consent was reached on all 221 patients and 37/221 (16,7%) subjects were rated as positive. Agreement between the final expert assessment and the NLT algorithm was 92% (Table I). A discrepancy was observed in 18 assessments (Table I). The proportion of female patients in the NLT-positive group (72%) reflected the proportion in the total PCS sample (69%).

**Table I:** Expert consensus with NLT algorithm

Factor	Expert consensus NLT positive	Expert consensus NLT negative
NLT Algorithm positive	32	13
NLT Algorithm negative	5	171

**Fig. 2:**
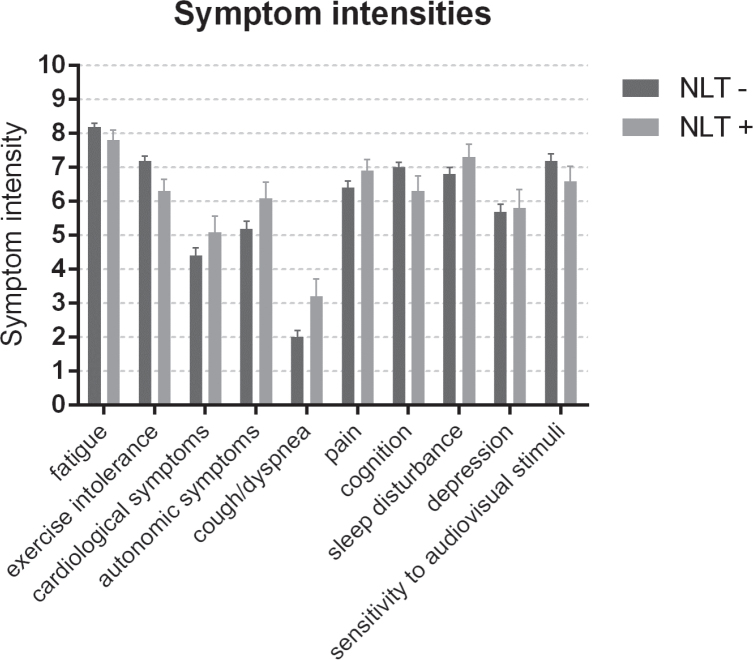
Self-reported symptom intensities of 221 PCS patients. Symptom intensity responses (mean, SEM) from PCS questionnaires for 36 NLT-positive and 173 NLT-negative PCS patients. Subjects reported multiple symptoms characteristic for PCS. Groups are not significantly different in any symptom at a family-wise error rate of 5%.

Mean self-reported PCS symptom intensities are presented in Fig. 2. For the questionnaire analysis, a symptom was defined as clinically relevant if it was reported as frequent, had not been present prior to SARS-CoV-2 infection, and had an intensity greater than 4 on the 10-point scale. All patients reported multiple symptoms typical of PCS (1). The most common symptoms were chronic fatigue, exercise intolerance, pain, sleep disturbances, and cognitive impairment. No significant differences in symptom profiles were observed between NLT-positive and NLT-negative patients (Welch test at family-wise error rate FWER 0.05). All patients were unable to work for more than 6 months.

## DISCUSSION

Consistent with previous publications, our findings indicate that a relevant proportion of patients with PCS present with disturbances in circulatory regulation compatible with POTS. In our cohort, 17% of patients fulfilled the expert criteria for this condition.

The prevalence of POTS in the general population has been estimated, largely based on clinical experience, to range between 0.2% and 1% in the United States (9). Our observed prevalence is therefore considerably higher than in healthy individuals, although somewhat lower than reported in studies investigating patients following viral infections, including PCS (3, 10,11). In our study, patients have been suffering from PCS for more than a year, so that a proportion of patients may have already partially recovered from autonomic dysfunction. Our NLT positive patients may either have persistent POTS – which may represent a distinct PCS subgroup – or may have developed POTS through a deconditioning effect after long-term reduced mobility.

In general, female patients are known to be more frequently affected by PCS (1), consistent with our findings of about 70% females in the PCS cohort as well as among POTS-positive patients.

Evaluation of the NLT algorithm showed very good agreement between expert raters. Minor discrepancies between experts were easily resolved through case-by-case discussion. Differences between expert assessment and algorithmic classification were mainly related to the time course of BP and HR, as well as unclear symptom reporting during the test. Our results suggest that the NLT algorithm may be a reliable tool for quantitative, semi-automatic POTS diagnosis.

In the literature, POTS patients have been reported to show similar symptoms to PCS patients (12, 13). Conversely, in our PCS patient cohort, the vast majority of patients also reported orthostatic symptoms, regardless of objective haemodynamic findings. Importantly, symptom profiles (see Fig. 2) did not differ between NLT-positive and NLT-negative patients. Therefore, questionnaires alone appear insufficient to replace objective diagnostic testing, and may overestimate the prevalence of autonomic dysfunction (14).

This highlights the need for objective diagnostic tools to guide targeted rehabilitation strategies. Both pharmacological and non-pharmacological interventions can effectively improve haemodynamic regulation (5, 15), e.g., using a ß-blocker, lower-body compression garments, or targeted muscle training.

In conclusion, quantitative assessment using the NLT represents a robust and clinically useful tool for evaluating circulatory dysregulation in PCS. Identification of PCS patients with autonomic dysfunction is a prerequisite of targeted rehabilitation strategies. The largely overlapping symptom profiles of NLT-positive and NLT-negative patients underline the need for routine objective testing.
